# Visual Task Demands and the Auditory Mismatch Negativity: An Empirical Study and a Meta-Analysis

**DOI:** 10.1371/journal.pone.0146567

**Published:** 2016-01-07

**Authors:** Stefan Wiens, Malina Szychowska, Mats E. Nilsson

**Affiliations:** 1 Gösta Ekman Laboratory, Department of Psychology, Stockholm University, Stockholm, Sweden; 2 Institute of Acoustics, Department of Physics, Adam Mickiewicz University, Poznan, Poland; University of Salamanca- Institute for Neuroscience of Castille and Leon and Medical School, SPAIN

## Abstract

Because the auditory system is particularly useful in monitoring the environment, previous research has examined whether task-irrelevant, auditory distracters are processed even if subjects focus their attention on visual stimuli. This research suggests that attentionally demanding visual tasks decrease the auditory mismatch negativity (MMN) to simultaneously presented auditory distractors. Because a recent behavioral study found that high visual perceptual load decreased detection sensitivity of simultaneous tones, we used a similar task (*n* = 28) to determine if high visual perceptual load would reduce the auditory MMN. Results suggested that perceptual load did not decrease the MMN. At face value, these nonsignificant findings may suggest that effects of perceptual load on the MMN are smaller than those of other demanding visual tasks. If so, effect sizes should differ systematically between the present and previous studies. We conducted a selective meta-analysis of published studies in which the MMN was derived from the EEG, the visual task demands were continuous and varied between high and low within the same task, and the task-irrelevant tones were presented in a typical oddball paradigm simultaneously with the visual stimuli. Because the meta-analysis suggested that the present (null) findings did not differ systematically from previous findings, the available evidence was combined. Results of this meta-analysis confirmed that demanding visual tasks reduce the MMN to auditory distracters. However, because the meta-analysis was based on small studies and because of the risk for publication biases, future studies should be preregistered with large samples (*n* > 150) to provide confirmatory evidence for the results of the present meta-analysis. These future studies should also use control conditions that reduce confounding effects of neural adaptation, and use load manipulations that are defined independently from their effects on the MMN.

## Introduction

The ability to focus on task-relevant events is critical for goal-directed behavior. However, this focus on task-relevant events increases the risk of missing events that may not be task-relevant but are still important. For example, while somebody is in a grocery store looking for apples, the person will respond to the ringing of his or her own mobile phone [1] and also to that of somebody else’s, despite the fact that responding is not relevant for purchasing apples. While responding to task-irrelevant environmental cues may not be useful in the context of ones’ goals, it is often unknown whether and when important information may be presented. Therefore, it seems beneficial to have a general system that continuously monitors the environment. Hearing seems particularly useful for this task, because unlike vision, is not restricted to a location in space.

To study this monitoring system in auditory perception, previous research has presented series of tones to examine whether the brain detects deviations in these tone series [2]. Most studies have employed oddballs: One tone is presented repeatedly (the standard) and is replaced occasionally (e.g., on 20% of trials) by another tone (the deviant). This deviation results in an auditory mismatch negativity (MMN) in the event-related potential (ERP). That is, in the difference ERP (computed as the ERP to deviants minus the ERP to standards), the auditory MMN represents a frontal-central negativity between 100 and 250 ms after tone onset. Two mechanisms for the MMN have been proposed: sensory memory and neural adaptation [3]. In the traditional view as sensory memory (or similar models such as regularity violation and predictive coding) (for review, see [3]), the MMN reflects the detection of the mismatch between the auditory sensory memory and the incoming deviant [2, 4, 5]. In terms of neural adaptation, the MMN reflects a less attenuated N1-response to the (infrequent) deviant than the (frequent) standard [6].

The degree to which the MMN is independent of attention has been debated for several decades [7, 8]. In most previous studies, subjects showed an auditory MMN even when they read a book or watched a silent movie [9–20]. In fact, current guidelines recommend recording the auditory MMN while subjects perform an interesting visual task (e.g., watch a silent movie) and are told to ignore task-irrelevant auditory stimuli [4]. However, because subjects may not strongly direct their attention on these tasks, the findings cannot be taken as strong evidence that the processing of task-irrelevant tones is independent of attention. Further, because in many studies, the visual stimuli for the main tasks and the tones were presented asynchronously [9–21], subjects may have shifted their attention between the visual stimuli of the main task and the tones.

In contrast, when subjects performed visual tasks that required them to attend closely to the visual stimuli, the auditory MMN to simultaneously presented task-irrelevant, auditory oddballs decreased (i.e., the MMN amplitude became less negative) in most studies [22–26], although one study reported no effect [27] and another study reported an increase of the MMN [28]. In all of these studies, participants performed demanding visual tasks that required continuous vigilance. Simultaneously, an oddball task was presented in which the task-irrelevant tones were either a standard tone (on most trials) or a deviant tone. Also, high and low visual demands were tested as different levels of the same task. Further, the trials were presented simultaneously with the visual stimuli to maximize competition between the task-relevant visual stimuli and the task-irrelevant tones. Thus, these studies had a stronger manipulation of attention than other tasks that permitted subjects to simply alternate their attention between the visual task and the tones [22]. Results for most studies suggest that when the demands of the visual task increased, the amplitude of the auditory MMN decreased [22–26]. These findings suggest that the processes that elicit the MMN require attentional resources. That is, processes of standard formation and/or deviance detection [3, 5, 29, 30] or neural adaptation [3, 6] are impaired by demanding visual tasks.

Despite the apparent decrease of the MMN by visual task demands, an MMN remains even under high visual demands [22–26]. For example, in a rapid serial visual presentation (RSVP) task, participants had to monitor rapid sequences (at 11.1 Hz) of letters for the occasional numeral [22]. During low visual demands, target numerals were shown at 100% contrast and distracter letters at 5% contrast (for 90 ms each). During high visual demands, all stimuli were shown at 5% contrast (for 20 ms each). The task-irrelevant tones (standard and deviant) were presented every fourth visual stimulus. Data were analyzed only for tones that preceded the first target numeral, while participants attended the letter stream. Results showed that during the visual task with high demands, the MMN decreased but was not eliminated. This attests to the robustness of the auditory MMN despite increases in visual task demands.

However, it may be that even stronger manipulations of visual attention might further reduce, if not eliminate the MMN. An extensive line of research has shown that perceptual load decreases processing of distracters [31]. According to load theory [32, 33], attentional resources are limited, and targets and distracters compete for attentional resources. If a main task consumes all of the available attentional resources (i.e., high perceptual load), then attention is not drawn to distracters and they are processed less.

In support of strong effects of perceptual load, high perceptual load decreased sensitivity (as indexed by *d′*) in detecting concurrent tones [34]. Participants performed a letter identification task (X or N) within a ring of six letters. During low perceptual load, the target letter was shown together with smaller Os, whereas during high perceptual load, the target letter was shown together with other letters (randomly drawn from the set of H, K, M, V, W, and Z). Simultaneous with the onset of the letter ring, a short tone was presented on some trials. Participants were instructed both to detect the tone and to identify the letter. In three experiments (conducted to rule out potential confounds), the sensitivity to detect tones decreased during high load. The authors concluded that these findings suggest that high perceptual load produces inattentional deafness.

Because previous behavioral research suggests that perceptual load decreases distracter processing [34], the current study used a similar task to study effects of perceptual load on the auditory MMN. As in the original study, participants performed a letter detection task with different levels of visual perceptual load while irrelevant tones were presented simultaneously with the onset of the letter ring. Although we tried to follow the design of the original study as much as possible, several modifications were implemented in order to optimize the task for MMN analysis. First, a tone (either standard or deviant) was presented on each trial. Second, the inter-trial interval (ITI) was reduced to 1 s (from 4.9 s) to increase the number of tone trials and thus, the signal-to-noise ratio for obtaining an MMN. Third, because of the short ITI (1 s), a simpler letter detection task (X) rather than the letter identification task (X or N) was used to ensure reaction times below 1 s (as revealed by pilot testing). Perceptual load was manipulated such that during low load, six identical letters were shown, and during high load, six different letters were shown. Fourth, tone intensity was increased to match intensities used in previous MMN studies in order to maximize sensitivity in obtaining an MMN and to permit comparison of the present MMN results with those in previous studies [22–28]. Fifth, participants were instructed to ignore the tones (rather than to detect the tones as in the original study). Taken together, our task was not identical to that of Raveh and Lavie but was adapted to optimize it for the measurement of the auditory MMN and to make its parameters comparable (i.e., tone on every trial, rather high tone intensity, and instructions to ignore the tones) to those used in previous MMN studies that manipulated visual task demands [22–28].

In sum, the present study examined the effects of visual perceptual load on the auditory MMN to task-irrelevant tones. Because perceptual load tasks decrease detection sensitivity to concurrent tones [34], we predicted that high perceptual load will reduce—if not eliminate—the auditory MMN. Although previous studies [22–26] suggested that demanding visual tasks decrease the MMN, we predicted that effects of perceptual load on the auditory MMN may be stronger than those of other manipulations.

## Materials and Methods

### Participants

Participants (*N* = 28; mean age = 25.0, *SD* = 3.3; 9 women) were students from local universities in Stockholm, Sweden. The study was approved by the Stockholm section of the Central Ethical Review Board in Sweden and was conducted in accordance with the guidelines in the Helsinki Declaration. All participants gave written informed consent, were debriefed after the experiment, and were compensated with one movie voucher.

### Materials and Procedure

**Fig 1:**
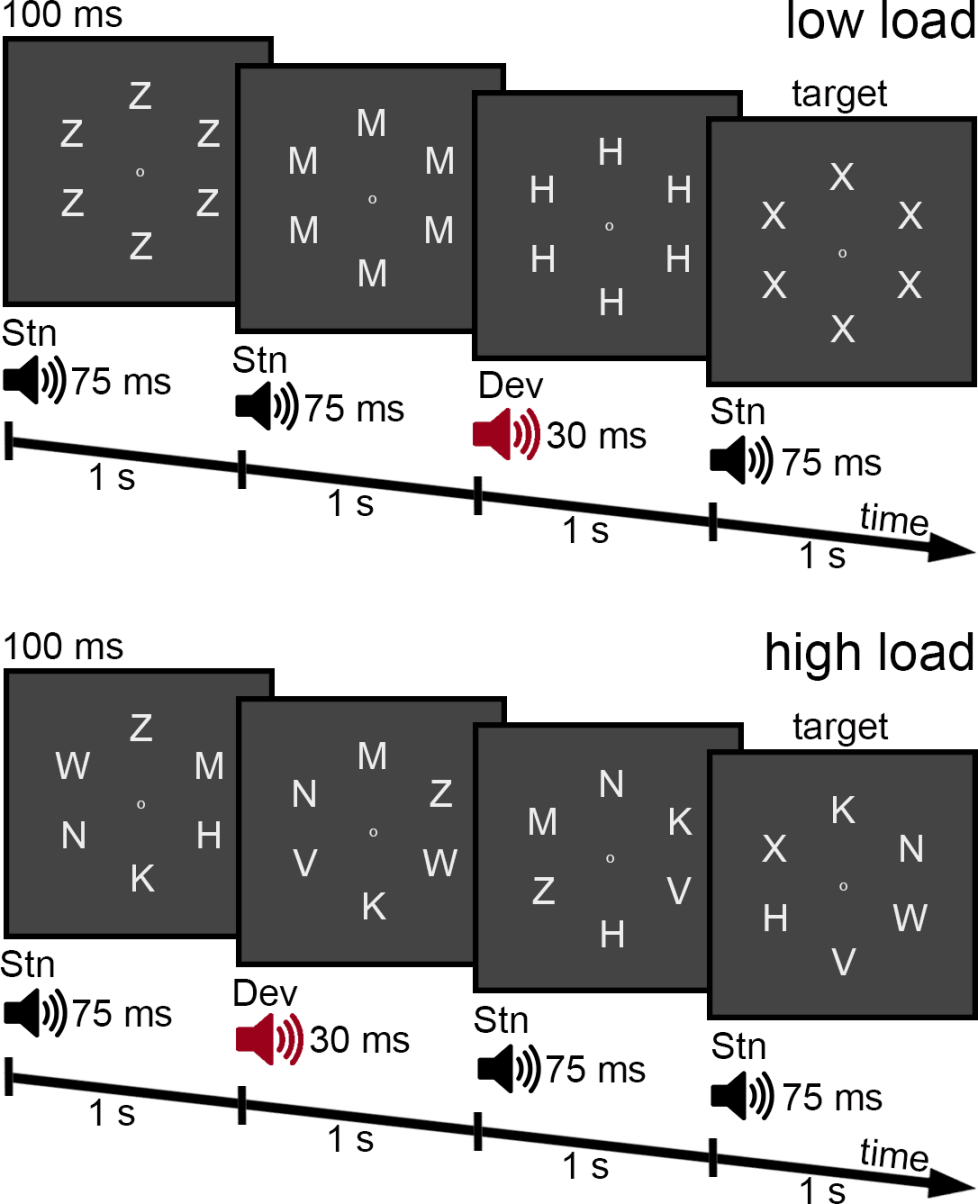
Illustration of the letter detection task during low load (top) and high load (bottom). Subjects had to respond when the letter ring included an X (target). Task-irrelevant complex tones (standard and deviants) were presented simultaneously with the onset of the letter rings. Stn = standard, Dev = deviant.

Participants performed a speeded letter detection task (detect X) and were instructed to push the space bar whenever an X was shown (20% of trials) (see Fig 1). On each 1-s trial, a 6-letter ring was shown for 100 ms. The 6 letters were shown at positions 2, 4, 6, 8, 10, and 12 o’ clock. A small dot was shown at the center of the letter ring throughout the experiment. Participants were instructed to keep their gaze on this dot while covertly attending to the 6 letters in the ring. The size of each letter was 0.53 x 0.53 degrees (visual angle), the distance between the centers of the letters was 0.98 degrees, and the diameter of the ring (for the centers of the letters) was 3.38 degrees. During low load, the 6 letters were identical, whereas during high load, the 6 letters were different. On each trial, letters were drawn randomly without replacement from the set of H, K, M, N, V, W, and Z. On 20% of the trials (response trials), the letter X was shown (six Xs during low load and one X together with 5 other letters during high load). For each set of five consecutive trials, a response trial occurred only during one of the first three trials (randomly determined). Thus, there were at least 2 nonresponse trials before the next response trial.

Participants performed 10 blocks of 250 trials each (i.e., 200 nonresponse trials and 50 response trials) with short breaks in between blocks. Between consecutive blocks, low and high load alternated. Each participant started with either low or high load (counterbalanced across participants). In total, participants performed 1000 nonresponse trials and 250 response trials for each load.

While participants performed the letter detection task, tones were presented simultaneously with the onset of the letter rings. Tones were presented with over-ear headphones (Sennheiser HD 280 Pro). Participants were instructed to ignore the tones. The standard tone (75 ms) and the deviant tone (30 ms) were complex tones with f0 = 500 Hz (higher harmonics at 1000 Hz and 1500 Hz with a drop of 3 dB/harmonic) at 76 dB SPL and with a 5 ms fade in and a 5 ms fade out. Over trials, 20% of tones were deviants during both response trials and nonresponse trials. The number of standard trials in between successive deviants ranged from 2 to 6 (mean = 4 trials). For the 1250 trials in each load, 800 trials were standards and 200 were deviants during nonresponse trials, and 200 were standards and 50 were deviants during response trials. The experiment was programmed in Presentation 14.8 (Neurobehavioral Systems, Albany, CA).

Before the first block, participants practiced the relevant task (low or high load) until they felt that instructions were clear and they were ready to start the main task. At the beginning of each block, between 5 to 8 nonresponse trials with standard tones were presented but were not analyzed.

### Electroencephalography

#### EEG recording

EEG data were recorded only from six electrodes at standard 10/20 positions (Fpz, Fz, Cz, M1, M2, and tip of nose) with an Active Two BioSemi system (BioSemi, Amsterdam, Netherlands). Fpz, Fz, and Cz were recorded with pin electrodes in a 64-electrode EEG cap; and M1, M2, and the tip of the nose were recorded with flat electrodes attached with adhesive disks. Two additional, system-specific electrodes were recorded with pin electrodes in the EEG cap. The CMS (between PO3 and POz) served as the internal reference electrode, and DRL (between POz and PO4) as the ground electrode. Data were sampled at 512 Hz and filtered with a hardware low-pass filter at 104 Hz. No high-pass filter was used. All physiological data was processed offline using the FieldTrip toolbox in MATLAB [35]. Continuous data were re-referenced to the tip of the nose.

#### ERP analysis

To minimize movement artifacts, ERPs were computed only for correct rejections (i.e., nonresponse trials without any button presses) (e.g., [36, 37]). Also, standard tones were excluded if they followed immediately after a deviant [38, 39]. Epochs were extracted from 100 ms before tone onset to 800 ms after. Each epoch was baseline corrected with the 100-ms interval before tone onset. For each participant, amplitude ranges within individual epochs were extracted, and the distribution of these amplitude ranges across epochs was inspected to exclude apparent outliers. This inspection was blind to the condition (i.e., tone deviance by load) of individual trials. To identify the MMN, a difference wave was computed as the ERP to deviants minus the ERP to standards; in this analysis, low and high load were collapsed. On the basis of visual inspection (see Results for more information), the MMN was defined between 160 and 220 ms after tone onset. For this interval, mean amplitudes were extracted for Fz and Cz for each condition. Before artifact rejection, at least 637 trials with standard tones and 190 trials with deviant tones were available for each load condition. After artifact rejection, at least 539 trials (i.e., more than 82.5%) with standard tones and 173 trials (i.e., more than 86.5%) with deviant tones were available. For completeness, we also analyzed the data on all trials (i.e., before artifact rejection, there were 1000 standard trials and 250 deviant trials for each load condition). Importantly, the results for the MMN matched those reported below. For example, for the mean MMN amplitudes (i.e., deviant minus standard) at Fz (with nose as reference) between 160 and 220 ms, the mean difference (low load minus high load) was 0.12 μV, 95% CI [-0.64, 0.89]. These findings suggest that our concern about movement artifacts was unnecessary. Nonetheless, we report results from our (a priori) data processing strategy.

### Data Analysis

In the behavioral analysis, responses faster than 200 ms were excluded. Because the task had a rapid pace (ITI of 1 s), we were concerned that responses faster than 200 ms may be late responses to the previous trial. A 200-ms interval rather than a shorter interval was chosen to ensure that all potentially late responses were excluded. If these late responses were included, then these responses would have artificially decreased performance, as a late response to a target trial would count as a false alarm (on the subsequent trial) as well as a miss (on the current trial). However, when we counted the number of such fast false alarms across all conditions for each subject, the maximum number was 7 (mean = 2.2 trials). Because the maximum number of potential false alarms was 1000, this number seems negligible. Although additional analyses showed that it did not matter for the results whether we included or excluded the fast responses, we report results after excluding responses below 200 ms. Hit rates and false alarm rates were computed for each condition (i.e., tone deviance by load). Signal detection analyses were performed to compute *d′* and *c* [40]. To avoid floor and ceiling effects on hit and false alarm rates, we added 0.5 trial in the numerator and 1 trial in the denominator [41].

Statistical analyses of our data followed recent guidelines [42, 43]. These guidelines advocate against null hypothesis significance testing in favor of estimation. In estimation, the data are used to obtain a point estimate (e.g., observed mean) and an interval estimate (i.e., 95% CI) of the best estimate of the true (population) effect size. Accordingly, the size of the 95% CI provides information about the precision of the estimation [42, 43]. Notably, the 95% CI must not be interpreted as the 95% probability that the obtained CI captures the actual effect size [44]. In practice, the 95% CI should be viewed as our best estimate of the true effect size, given the present data [43]. This estimate may then be evaluated in relation to previous findings by integrating all studies in a meta-analysis [42]. Strictly speaking however, this interpretation of CI in terms of precision and likelihood is formally incorrect [45]. But, when *t* tests are used to evaluate mean differences (as used here), the formally correct *likelihood interval* (size 1/8) is identical to a 96% CI [46]. For simplicity and to follow current guidelines, we report the 95% CIs but discuss them in terms of likelihood intervals (i.e., the range of values that fit best in predicting the data).

## Results

Table 1 shows means (and *SD*s) for behavioral performance and mean amplitudes for the four conditions formed by combining the independent variables tone deviance (standard vs. deviant) and perceptual load (low vs. high). Table 2 shows the results of the repeated-measures ANOVAs for the main effects (i.e., deviance and load) and their interaction. The table also shows each effect size expressed as a mean difference (*M*_diff_) together with the two-tailed 95% CIs [47]. The interaction between load and tone deviance was captured by the difference score between difference scores [48]. That is, within each load, a difference score was computed as responses to deviants minus responses to standards. Then, another difference score was computed as the difference score for low load minus the difference score for high load.

**Table 1:** Means (and *SD*s) for behavioral performance and mean amplitudes for the four conditions (deviance by load).

Variable	Low loadstandard		Low load deviant		High loadstandard		High loaddeviant	
	*M*	*SD*	*M*	*SD*	*M*	*SD*	*M*	*SD*
*d′*	4.89	0.49	4.70	0.40	3.41	0.87	3.32	0.86
*c*	0.25	0.20	0.28	0.22	0.87	0.39	0.74	0.42
RT hits (ms)	447.80	37.79	443.06	36.28	558.62	60.75	552.67	67.23
Mean amps (μV)								
Fz (nose)	1.89	2.25	-0.04	2.36	0.62	2.14	-1.27	2.22
Cz (nose)	1.74	2.36	0.17	2.43	-0.11	2.02	-1.34	2.20
Mastoids (nose)	-1.55	1.88	-0.49	1.99	-1.99	1.47	-1.07	1.29
Fz (mastoids)	3.44	2.71	0.45	2.21	2.62	2.71	-0.21	2.38
Cz (mastoids)	3.29	2.76	0.66	2.29	1.88	2.55	-0.28	2.21

**Table 2:** Results of repeated-measures ANOVAs with the independent variables Deviance (deviant minus standard), Load (low load minus high load), and their interaction on behavioral performance and mean amplitudes.

Variable	*F*	*p*	*M*_diff_	95% CI	
				Lower	Upper
*d′*					
Deviance	8.23	.008	-0.14	-0.24	-0.04
Load	107.66	< .001	1.44	1.15	1.72
Dev x Load	0.96	.335	-0.10	-0.29	0.10
*c*					
Deviance	3.61	.068	-0.05	-0.11	0.01
Load	48.33	< .001	-0.53	-0.69	-0.38
Dev x Load	4.54	.042	0.16	0.01	0.31
RT hits (ms)					
Deviance	6.78	.015	-5.34	-9.55	-1.13
Load	213.38	< .001	-110.22	-125.70	-94.74
Dev x Load	0.07	.788	1.22	-8.00	10.43
Fz (nose)					
Deviance	57.39	< .001	-1.91	-2.43	-1.39
Load	19.38	< .001	1.25	0.67	1.83
Dev x Load	< 0.01	.952	-0.03	-0.91	0.85
Cz (nose)					
Deviance	24.33	< .001	-1.40	-1.98	-0.82
Load	36.99	< .001	1.68	1.12	2.25
Dev x Load	0.69	.412	-0.33	-1.16	0.49
Mastoids (nose)					
Deviance	35.81	< .001	1.00	0.65	1.34
Load	4.40	.045	0.51	0.01	1.01
Dev x Load	0.18	.676	0.14	-0.55	0.84
Fz (mastoids)					
Deviance	126.63	< .001	-2.91	-3.44	-2.38
Load	10.48	.003	0.74	0.27	1.21
Dev x Load	0.51	.481	-0.17	-0.66	0.32
Cz (mastoids)					
Deviance	71.29	< .001	-2.40	-2.98	-1.81
Load	25.84	< .001	1.17	0.70	1.65
Dev x Load	4.47	.044	-0.48	-0.94	-0.01

For all ANOVAs, *F*(1, 27). Deviance refers to the main effect of deviant minus standard, Load refers to the main effect of low load minus high load, and Dev x Load refers to their interaction (i.e., [deviant–standard for low load]–[deviant–standard for high load]). *M*_diff_ is the mean difference score of the comparison. It expresses the comparison in its original units (μV) and thus, is an unstandardized effect size measure of the comparison of interest.

### Behavior

As shown in Table 1 and confirmed by the repeated-measures ANOVAs (Table 2), compared to high load, low load showed better letter detection performance (i.e., larger *d′* and shorter RTs to hits). Also, compared to standard tones, deviant tones lowered performance slightly (i.e., lower *d′*). Participants were generally more conservative (i.e., *c* was more positive) during high load than low load.

### Event-related potentials (MMN)

**Fig 2:**
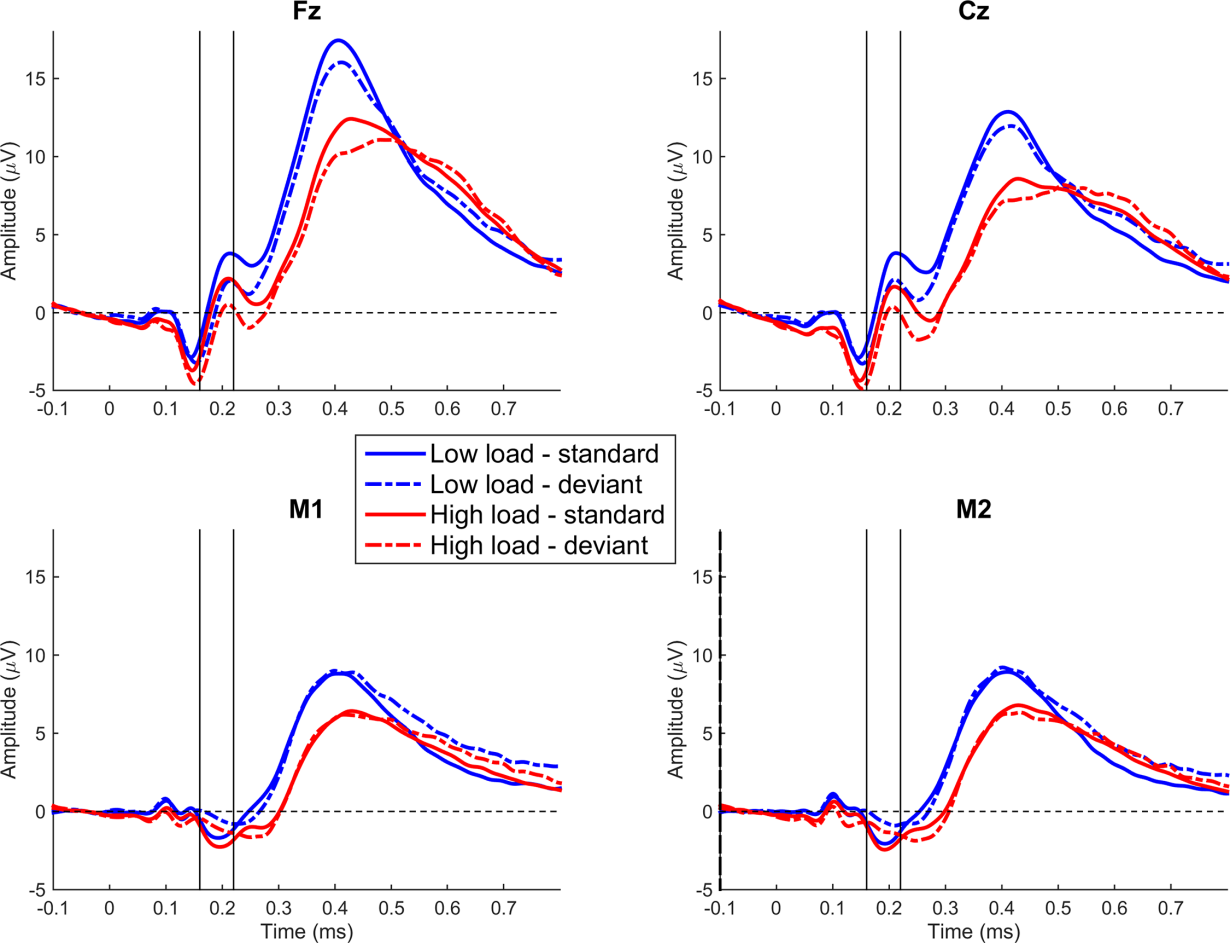
Grand mean (*N* = 28) ERP waves (referenced to tip of nose) after tone onset for the relevant electrodes, separately for the four conditions (Deviance x Load). For visualization purposes only, the ERPs were low-pass filtered (30 Hz, 6 dB roll-off, Butterworth). The interval between 160 and 220 ms after tone onset was used to capture the mismatch negativity.

Fig 2 shows mean ERP waves for the relevant electrodes, separately for the four conditions (i.e., Deviance x Load). In the comparison of deviants versus standards at around 200 ms after tone onset, the ERPs are relatively negative for deviants versus standards at Fz and Cz (top row) and relatively positive at the mastoids (bottom row). This relative negativity at Fz and Cz with a polarity reversal at the mastoids is a central feature of the MMN (for review, see [4]). To isolate the MMN for each load condition, the difference waves were computed as deviants minus standards at each level of load.

**Fig 3:**
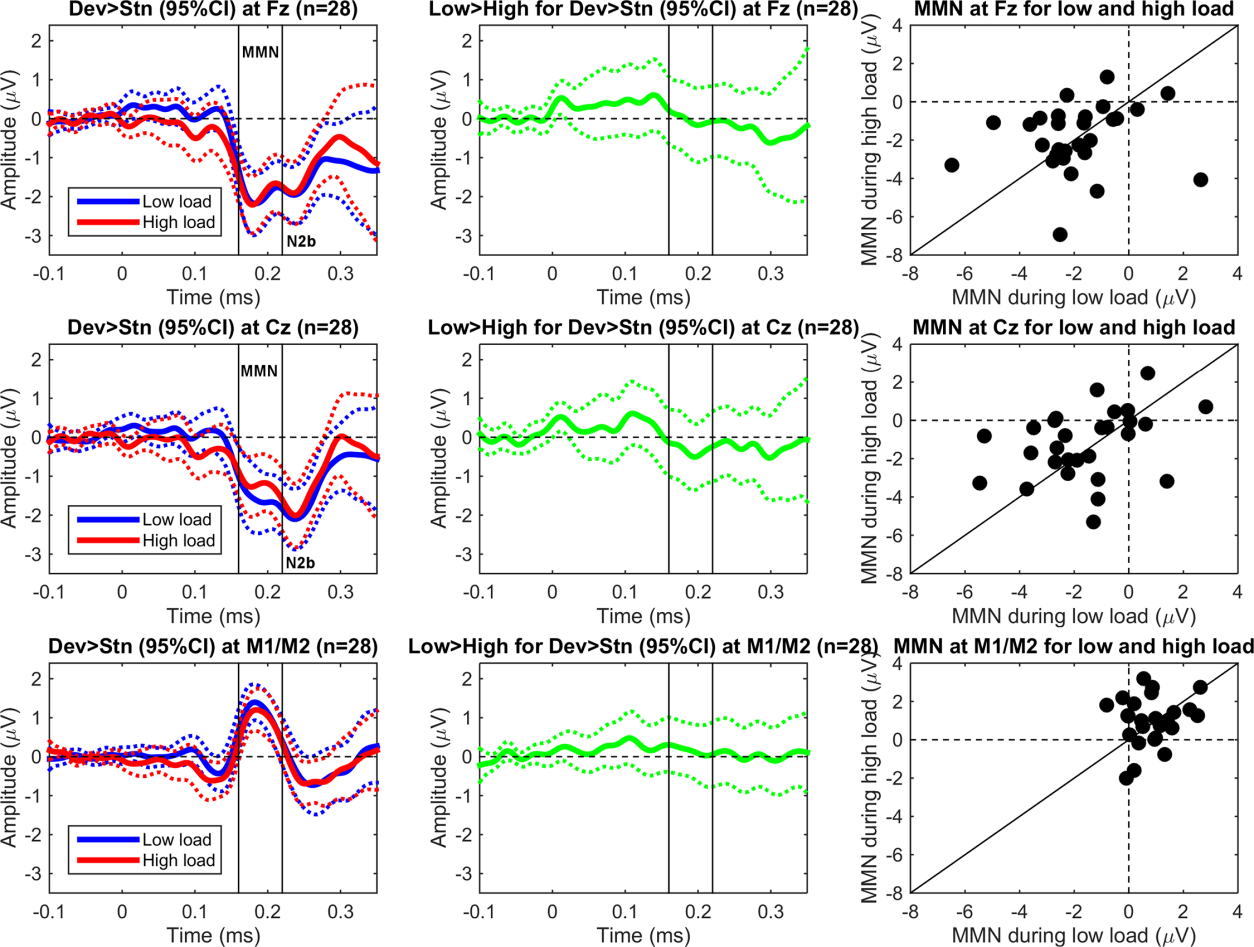
Grand mean (*N* = 28) ERP difference waves (deviant minus standard, referenced to tip of nose) after tone onset at Fz (top), Cz (middle), and mastoids (bottom) for low and high perceptual load (left), the subsequent difference of low load minus high load (middle), and a scatterplot of individual mean MMN amplitudes (i.e., deviant minus standard) for low and high load (right). For visualization purposes only, the ERPs were low-pass filtered (30 Hz, 6 dB roll-off, Butterworth).

Fig 3 shows the ERP difference waves (deviants minus standards, referenced to the tip of the nose, with 95% CI) at Fz (top), Cz (middle), and mastoids (bottom) for low and high load (left), the subsequent difference waves between load conditions (middle), and a scatterplot of mean amplitudes (160–220 ms) for low and high load across individuals (right). As shown in the left panel, the MMN was apparent around 200 ms in the negative wave for Fz and Cz (top and middle) and in the positive wave for the mastoids (bottom). In the present analyses, the MMN was defined in terms of the first peak (160 to 220 ms).

Note that Fz and Cz also tended to show a second negative peak at around 240 ms (see Fig 3). Because the mastoids did not show such a second peak, this second peak is probably an N2b [49]. The N2b peaks about 250–300 ms after tone onset is often observed when participants attend overtly to the stimuli, but it can also be observed for covert attention [50]. Thus, the observation that visual task performance was lower when deviants were presented (see Tables 1 and 2) is consistent with the idea that the deviants captured covert attention. Critically, only the first peak showed a polarity reversal over the mastoids; this is typically considered to be an indicator for the genuine MMN [49]. Further note that no P3a was apparent; that is, there was no relative positivity for deviants versus standards at either Fz or Cz about 250 ms after tone onset (e.g., Zhang et al., 2006).

As shown in the left panel and also in the middle panel (i.e., the difference wave between the two load conditions), the MMN was comparable for the two load conditions. That is, the ERP difference wave between high and low load was centered on zero (middle panel), and individual mean MMN amplitudes for low and high load were distributed symmetrically around the central diagonal (right panel).

**Fig 4:**
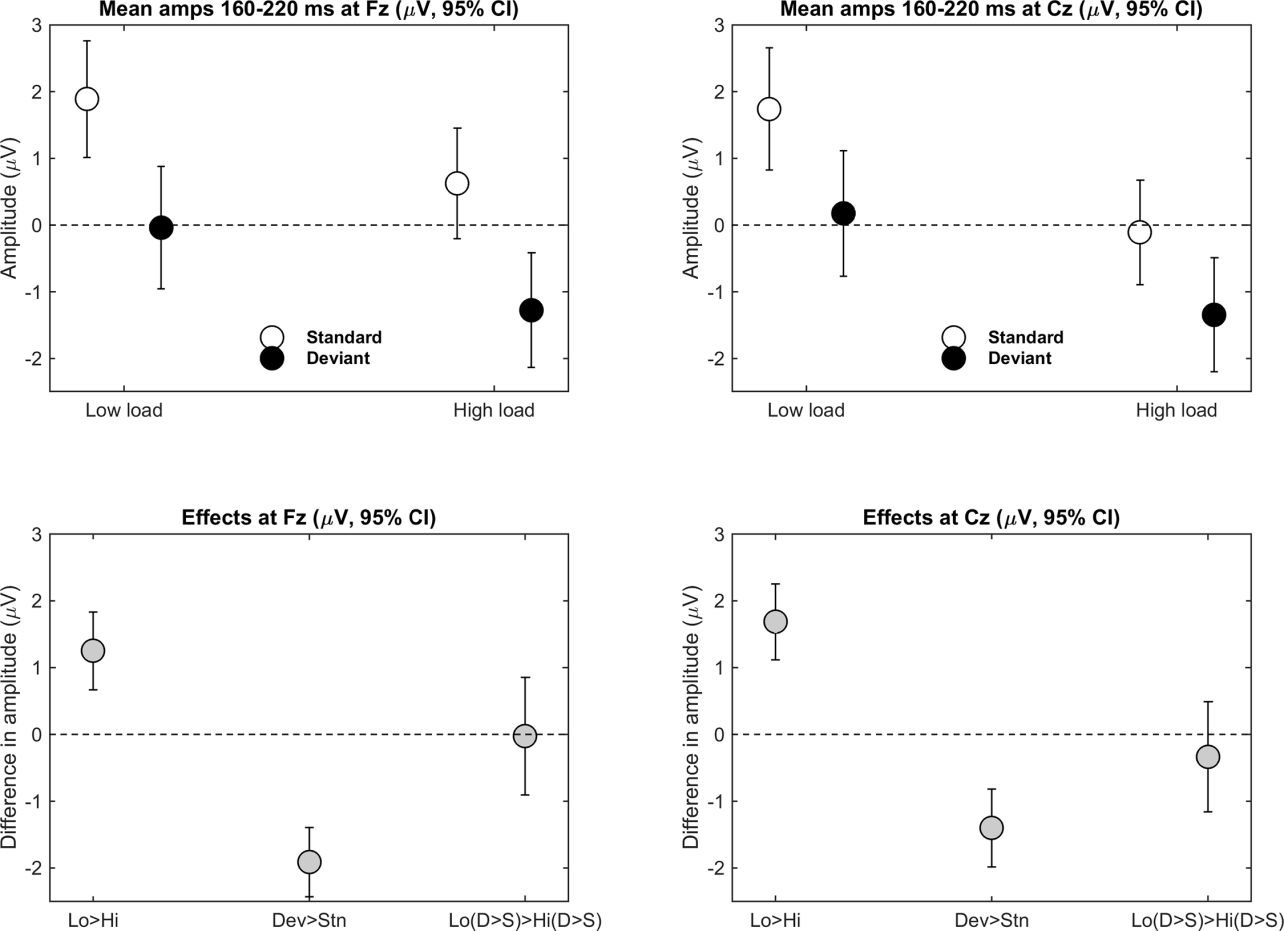
Mean amplitudes (referenced to tip of nose) for the four conditions (Deviance x Load) across 160–220 ms after tone onset at Fz (left) and Cz (right). The top row shows condition means (95% CI for each variable), and the bottom row shows mean difference scores (95% CI for the differences) between conditions for effects of load (low minus high), tone deviance (deviant minus standard), and the interaction (i.e., [deviant–standard at low load]–[deviant–standard at high load]).

Fig 4 shows mean amplitudes (referenced to the tip of the nose, with 95% CI) between 160–220 ms after tone onset for the four conditions (tone deviance by load) at Fz (left) and Cz (right). As shown in the bottom row, although there was clear evidence for an overall MMN (i.e., deviant minus standard was negative), there was no evidence for an interaction between load and deviance, suggesting that the MMN was comparable during low and high load. As shown in Tables 1 and 2, mean amplitudes for Fz and Cz and the mastoids (when referenced to the tip of the nose) provided no evidence for an interaction between tone deviance and load. For completeness, we also analyzed effects on Fz and Cz when referenced to the mastoids [23, 24]. Because of the polarity reversal of the MMN over the mastoids or ears, the mastoids or ears may be used as a reference to maximize sensitivity (for review, see [4]). Although no interaction was observed for Fz, results provided some evidence for an interaction at Cz. That is, the MMN was relatively more negative during low load (-2.64 μV) than during high load (-2.16 μV), and this difference (-0.48 μV) had a 95% CI of -0.94 and -0.01 (this CI did not include zero, and the effect was thus, significant at *p* < .05).

## Discussion

The present study used a perceptual load task with visual stimuli that substantially decreased behavioral detection sensitivity to simultaneous, task-irrelevant tones [34]. Therefore, we predicted that this perceptual load would reduce the auditory MMN to simultaneous, task-irrelevant tones. Results showed that although high perceptual load decreased task performance (lower *d′* and longer RTs to hits), a robust MMN was observed during both low and high perceptual load with no convincing evidence for differences between low and high load.

The present study found an MMN at Fz and Cz (with a polarity reversal at the mastoids) during both low and high perceptual load. However, there was no clear evidence that perceptual load decreased the MMN. These results were obtained when the MMN was measured with the tip of the nose as a reference, as recommended [4]. We also measured the MMN at Fz and Cz referenced to the mastoids to maximize the size of the MMN (for review, see [4]). When referenced to the mastoids, the MMN at Cz (but not at Fz) showed a significant effect of load (*p* < .05) in that the 95% CI did not include zero (-0.94 to -0.01 μV). However, if visual demands actually changed the MMN by as little as -0.01 μV, then this effect would seem to be as meaningful as no change. Because these findings are tentative at best and were not obtained at the main electrode (i.e., Fz), these findings do not provide convincing evidence that the MMN decreases during high perceptual load.

Whereas perceptual load did not seem to affect the MMN, it had large effects on behavioral performance. High perceptual load decreased detection sensitivity (by about 1.5 *SD*) and increased reaction times to hits (by about 110 ms). Notably, Table 1 suggests that across loads, detection performance decreased slightly when the concurrent tone was a deviant rather than a standard. This observation matches results for an MMN at both levels of load in that the tones were apparently processed irrespective of load.

One possible argument for the apparent absence of effects of perceptual load on the MMN is that the present level of high perceptual load was not high enough to observe effects on the MMN. Indeed, it is possible that a stronger manipulation of perceptual load might result in clearer effects of perceptual load on the MMN. Unfortunately, a general criticism of load theory [32, 33] is that there is no independent criterion for a high level of perceptual load [51]. As such, the reasoning for effects of load risks becoming circular: If there is an effect of load, then the level of load was high enough. If there is no effect of load, then it was not high enough [52]. In this study, we used a task that clearly decreased sensitivity (as indexed by *d′*) in detecting concurrent tones [34], and participants in our study were instructed to ignore the tones. Thus, it seemed reasonable to expect similar (or at least any) effects of perceptual load on the MMN. Also, although we used louder tones than Raveh and Lavie, the present tone intensity was comparable to that used in previous studies on effects of demanding visual tasks on the MMN [22–28]. Thus, the present findings for perceptual load can be readily compared with findings obtained with other manipulations in this previous MMN research.

Another possible argument for the present findings may be that our main electrodes were positioned only at midline (Fz, Cz) and were thus, insensitive to potential laterality effects. However, laterality effects seem to be most apparent for changes in speech stimuli (phoneme changes) [2] and for laterally presented stimuli [53]. Because we used bilateral tones that differed only in duration, laterality effects seem unlikely. In support, similar studies in this field included lateral electrodes but apparently found effects only at midline electrodes [22, 24–28]. So, we chose only Fz and Cz and the mastoids because the MMN is captured well by these electrodes (for review, see [4]). Also, because of limited resources, we decided to use fewer electrodes but run more subjects (our study included 28 subjects whereas previous studies had a mean of 13 subjects, range = 10 to 20).

In terms of null-hypothesis significance testing, the present findings represent so-called null findings in the main analyses (because *p* > .05). However, our study design was appropriate, the study was carefully conducted, and our results showed clear evidence for an overall MMN. Therefore, the present results are valid findings. As such, these results should increase our understanding of the effects of visual demands on the MMN by prompting two questions. First, do the present findings show that the effect of perceptual load on the MMN is smaller than that of other, similar visual task demands? According to null-hypothesis significance testing, it may be argued that our study did not find significant effects whereas most previous research apparently did [22–26]. Thus, we should examine why our study apparently did not find any effect. For example, is it possible that we did not observe an effect because we used duration deviance rather than frequency deviance [22–26]? Although this reasoning has a long tradition in Psychology, it is problematic because even exact replications of studies that sample from the same population can be expected to vary because of sampling error [43]. If so, the present findings may not be surprising or noteworthy because they might simply reflect chance variability between studies. Simply put, because of chance, the observed effect sizes in different studies will be bigger or smaller than the true effect size. Thus, it may well be that our study just happened to sample at the lower end of this range. As described below, a heterogeneity analysis within a meta-analysis can address quantitatively if the variability in effect sizes between studies is likely due to chance (i.e., all studies sample from a single effect size population) or if there is reason to believe that there is systematic variability (heterogeneity) between studies (i.e., different effect size populations are sampled). Notably, we originally proposed that the auditory MMN would be reduced more by perceptual load than by other visual task demands. A meta-analysis would also be useful to address this question. Specifically, the meta-analysis should show evidence for systematic variability (heterogeneity) between studies with a stronger decrease of the MMN from perceptual load than other visual tasks. However, the present null findings seem to suggest that if anything, perceptual load had either a weaker or similar effect on the MMN compared to other tasks. The heterogeneity analysis within the meta-analysis will be helpful in distinguishing between these two possibilities.

Second, do the present null findings challenge the idea of effects of visual task demands on the MMN? Because Psychology as a discipline has traditionally published only significant findings, this publication bias produces effect sizes that are overestimated [54–56]. Accordingly, does the combined evidence across the present findings and previous studies with other visual demanding tasks support the conclusion that the MMN decreases during increased visual task demands?

In sum, a meta-analysis would be useful to quantitatively determine whether or not the present findings differ systematically from previous results, and whether or not the evidence for an effect is changed when present and previous findings are combined [43, 57, 58]. To address these questions, we performed a meta-analysis of the present results together with previous studies that used a similar set-up [22–28].

### Meta-analysis

We conducted a meta-analysis (with the ESCI software in [43]) of the effects of visual task demands on the MMN in the EEG. Inspired by an estimation approach [42, 43], we attempted to quantitatively integrate our findings with those of previous studies with meta-analytic tools. Meta-analysis is often viewed as a comprehensive quantitative integration of numerous studies on a rather wide topic. However, meta-analytic tools are already useful to combine results of at least two studies [43]. Thus, our selective meta-analysis should not be seen as an attempt to review all available evidence on effects on the MMN, but instead an attempt to quantitatively integrate only previous, published studies that closely resembled the present study design. Thus, this meta-analysis was highly selective in that published studies were included only if they used EEG, the visual task demands were continuous and varied between high and low load within the same task, and the task-irrelevant tones were typical standards and deviants (i.e., oddballs) that were presented simultaneously with the visual stimuli. Accordingly, studies were excluded for the following reasons (but are mentioned only once even if they fulfilled several criteria). Studies with other measures such as steady-state evoked potentials, MEG, or fMRI [59–62], studies in which visual and auditory stimuli were presented asynchronously [9–21, 63, 64], studies in which subjects had to read (see also [22]) or perform a working memory task [15, 18, 64–66], studies in which different tasks rather than task levels were compared [17, 67–69], studies with patterned auditory sequences rather than typical oddballs [70], and studies of clinical populations [69, 71] or altered states of consciousness [72, 73].

Because results for electrode Fz were reported in all articles, this meta-analysis focused on mean amplitudes at Fz, consistent with current guidelines [4]. The MMN amplitude at Fz was recorded between about 100 to 250 ms after stimulus onset and with a reference on the mastoid or ear [23, 24], tip of the nose [22, 26–28], or the chin [25]. Thus, this meta-analysis quantifies the published evidence for effects of visual task demands on the auditory MMN in studies that closely resembled the present study design.

**Fig 5:**
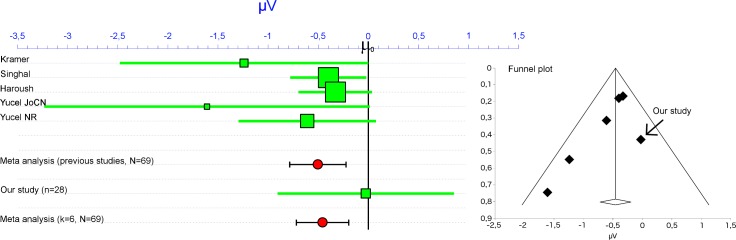
Meta-analysis of how visual task demands changed the MMN (forest plot on left and funnel plot on right). The analysis focused on the mean amplitudes at Fz (between about 100 and 250 ms after stimulus onset) and was expressed in μV (with 95% CI). The overall negative effect shows that the MMN (i.e., deviant minus standard) was more negative during low than high visual demands (i.e., MMN during low demands minus MMN during high demands). See main text for references to the individual studies. In the forest plot (left), the size of each square corresponds to the weight in the meta-analysis.

The first meta-analysis combined five studies [22–26]. Although two more studies fit our selection criteria [27, 28], they were excluded in this first meta-analysis because the predictions were unclear, as described further below. Table 3 shows the main statistics that were used for the meta-analysis, and Fig 5 shows a forest plot. In regards to the first five studies, one study [23] reported only the inferential statistics of the overall ANOVA with three conditions (i.e., for baseline, low load, and high load; with *F* = 4.6 and *p* < .05) and did not report inferential statistics for the specific comparison between low and high load. Although the study reported means and standard deviations (see Table 4 in [23]), the standard deviations refer to the variability within each condition and not to the variability of the difference scores between conditions. However, this variability of difference scores between conditions is critical in order to conduct inferential tests in repeated-measures designs [43, 74, 75]. In an attempt to estimate these data, we generated random data that matched the reported descriptives for the three conditions (see Table 4 in [23]). This approach worked well to match results for another study [28], as described below. In contrast, for 20,000 randomly generated data sets that matched the descriptives for the three conditions in the study [23], the minimum *F* value was 9.5 and the maximum *p* value was .003 (after Greenhouse-Geisser correction). Because we could not simulate matching data, we simply assumed that the specific contrast between low and high load was significant at *p* = .05.

**Table 3:** Statistics for amplitudes at Fz used in the meta-analysis.

Study	*N*	Low load (μV)	High load (μV)	Mean Diff (low-high)	*SD* Diff	*t*	95% CI	
							LL	UL
Kramer et al. (1995)	10	-1.05	0.19	-1.24	1.73^a^	-2.26^a^	-2.48^a^	-0.01^a^
Singhal et al.(2002)	20	-2.80	-2.40	-0.40	0.81	-2.20	-0.78	-0.02
Haroush et al. (2010)	13	-2.20	-1.87	-0.33	0.61	-1.96	-0.70	0.04
Yucel et al. (2005, JoCN)	13	-2.67	-1.06	-1.61	2.69	-2.16	-3.23	0.01
Yucel et al. (2005 NR)	13	-2.75	-2.14	-0.61	1.13	-1.94	-1.30	0.07
Muller-Gass et al. (2007)^a^	9	-	-	0.00	1.51	0.00	-1.16	1.16
Zhang et al. (2006)	11	-1.18	-2.10	0.92	0.79^a^	3.84^a^	0.39^a^	1.45^a^
Present study	28	-1.93	-1.90	-0.03	2.27	-0.06	-0.91	0.85

^a^Values were estimated.

In the meta-analysis with five studies [22–26] (see Fig 5), a random effects analysis (*k* = 5, *N* = 69) showed that the MMN amplitude (i.e., deviant minus standard) was larger (i.e., more negative) during low than high visual demands. That is, for the comparison of MMN during low load minus MMN during high load, the mean amplitude difference = -0.50 μV, 95% CI [-0.79, -0.22]. Notably, there was no evidence for heterogeneity among the effect sizes, *Q*(4) = 5.34, *p* = .25. The proportion of total variance that reflects variation in true effect size was estimated to be 25% (*I*^2^ = 25.06%). Therefore, evidence from previous studies suggests that visual task demands decrease the auditory MMN.

Next, we included our results in the meta-analysis (Fig 5). Overall results of a random effects analysis (*k* = 6, *N* = 97) confirmed that the MMN was more negative during low than high visual demands, mean difference = -0.46 μV, [-0.72, -0.20]. There was still no evidence for heterogeneity among the effect sizes, *Q*(5) = 6.28, *p* = .28. The proportion of total variance that reflects variation in true effect size was estimated to be 20% (*I*^2^ = 20.42%). Thus, the present findings fit in well with those of previous research [22–26].

In the meta-analyses above, we excluded two studies with visual tracking tasks that involved moving circles [27, 28]. In these studies, several moving circles were shown and subjects had to track a subset of these circles while remembering which circles to track. This task differs from that of the other studies [22–26]. In these previous studies, subjects monitored a radar screen to detect and identify aircrafts during either low or high target density [23], landed an aircraft while ignoring tones during either low or high turbulence levels [24], used a joystick to center a moving cursor that changed in velocity (low demand) or acceleration (high demand) [25, 26], or monitored a rapid visual stream of letters for two numerals with the stimuli at either low demands (high contrast, long duration) or high demands (low contrast, short duration) [22]. In contrast to these tasks, the visual tracking task with moving circles required subjects to remember which circles to track and to track several circles at the same time during high visual demands.

In one study [28], participants had to track continuously one, three, or five moving circles (out of ten) while irrelevant tones were presented. In contrast to previous research, findings suggested that high task demands increased (rather than decreased) the MMN. That is, the mean MMN amplitudes at Fz were -1.18 μV while tracking one circle, -1.43 μV while tracking three circles, and -2.10 μV while tracking five circles. Thus, the MMN amplitudes increased (i.e., became more negative) with the number of tracked circles. The authors reasoned that in contrast to previous studies, their visual tracking task loaded visual spatial working memory and that this increase in the MMN fits with predictions for cognitive load rather than perceptual load. Specifically, whereas perceptual load should decrease distracter processing, cognitive load should increase distracter processing [33]. However, recent evidence suggests that visual spatial working memory has the same effects as perceptual load [76–79]. Accordingly, load theory would seem to predict that the visual tracking task (similar to perceptual load) should decrease the MMN rather than increase the MMN, opposite to the actual findings with the visual tracking task. Because predictions for the visual tracking task are mixed, we excluded this study in the above meta-analyses. Similarly, the other study [27] was excluded in the above meta-analyses because it used a similar visual tracking task. Participants tracked either one (of two) moving circles or two (of four) moving circles.

For completeness, however, these two studies were added in the next meta-analysis [27, 28]. Because one study [27] did not report enough data to compute the relevant statistics, the mean effect of load on the MMN was estimated as zero and the *SD* of the difference scores was estimated as 1.51 (i.e., the pooled *SD* of the difference scores for the five published studies in the first meta-analysis). For the other study [28], the effect of load was defined in terms of the difference between tracking one versus five circles. Thus, the mean difference of the MMN was 0.92. However, as for another study discussed above [23], the authors reported inferential statistics only for the overall ANOVA with three conditions (i.e., tracking 1, 3, or 5 circles, *F* = 5.02, *p* < .03 after Greenhouse-Geisser correction) and not the specific comparison between one and five circles. To estimate inferential statistics of this specific comparison, we simulated data for the three conditions with matching descriptives (see Table 4 in [28]). From 20,000 simulations, we selected the data set that had the closest match with an overall *F* value of 5.02 and *p* = 0.029 after Greenhouse-Geisser correction. Then, results of a paired *t* test between the conditions with one and five circles were used in the meta-analysis (see Table 3). The estimated *SD* of the difference scores was 0.79. After including these additional studies [27, 28], results of a random effects analysis suggested heterogeneity among the effect sizes (*k* = 8, *N* = 117), *Q*(7) = 32.85, *p* < .001. The proportion of total variance that reflects variation in true effect size was estimated to be 79% (*I*^2^ = 78.69%). Importantly, it appeared that only the study that found an increase in MMN contributed to this heterogeneity [28]. When this study was excluded (*k* = 7, *N* = 106), the heterogeneity was small, *Q*(6) = 6.98, *p* = .32, *I*^2^ = 14.05%. For these remaining seven studies, the MMN was more negative during low than high visual demands, mean difference = -0.43 μV, [-0.67, -0.18]. These findings confirm the usefulness of the meta-analysis because it provides quantitative evidence that a different population effect was sampled in one study [28] than in the remaining studies.

## General Discussion

The present study found a MMN at Fz and Cz (with a polarity reversal at the mastoids) during both low and high load, but the MMN did not differ between low and high load. We performed a meta-analysis to assess how the present results fit in with the overall evidence for an effect of demanding visual tasks on the MMN. This meta-analysis did not provide any evidence that the effect of perceptual load on the MMN in the present study differed systematically from that observed in previous tasks [22–26]. Such systematic variability would have been expected if the present perceptual load task was less (or more) effective than other tasks in decreasing the MMN. Accordingly, the present findings do not suggest that perceptual load differed systematically from (i.e., was less effective than) other demanding visual tasks in its effect on the MMN. Further, this lack of variability among effect sizes suggests that the choice of reference electrode may be irrelevant, as well as the choice of using a duration deviant (in the present study) rather than a frequency deviant (as in previous studies).

The meta-analysis further allowed us to assess the combined evidence for effects of visual task demands on the MMN. As shown in Fig 5, the meta-analysis of all available evidence (*k* = 6, *N* = 97) suggested that demanding visual tasks decrease the MMN. So, the combined evidence that includes our findings supports the idea that demanding visual tasks decrease the auditory MMN to simultaneous distracters. Therefore, visual task demands disrupt the process of sensory memory by either impairing standard formation and/or deviance detection [5, 30, 80], or the process of neural adaptation [6].

However, the present meta-analysis may overestimate the true effect size. Because of publication bias, studies have traditionally been published in Psychology only if they reported significant findings [54–56]. Because significant findings tend to have larger effect sizes than non-significant findings [81–83], the present meta-analysis may overestimate the true effect. Although tentative, the funnel plot in Fig 5 suggests that there may be a publication bias because the funnel lacks studies in the lower right corner of the funnel. This corner would show studies that typically would not be statistically significant because they have smaller (or even positive) effect sizes and larger sampling error. This observation could not be tested formally because recommendations for common tests of funnel asymmetry require at least 10 studies [84]. However, to reduce the possible effects of publication bias, one approach is to reduce the asymmetry by excluding studies that contribute most to the asymmetry [85]. When the two studies on the lower left-hand side of the funnel plot were excluded [23, 25], there remained an overall effect on the MMN. Before excluding these two studies (*k* = 6, *N* = 97), mean difference = -0.46 μV, [-0.72, -0.20], and after excluding these two studies (*k* = 4, *N* = 74), mean difference = -0.37 μV, [-0.59, -0.15]. Indeed, this limited influence of these two studies is already apparent in the forest plot of the meta-analysis (Fig 5), as the small size of the dots shows that their relative contribution to the overall effect is small. Thus, even if publication biases are considered by excluding two studies, the remaining evidence suggests that demanding visual tasks decrease the MMN to simultaneous auditory distracters. However, the credibility of this conclusion is discussed further below under limitations.

In the meta-analysis, we excluded two studies with a visual tracking task [27, 28] because the predictions were not entirely clear. The authors in one study [28] reasoned that because their visual tracking task loaded visual spatial working memory, this cognitive load should increase distracter processing [33]. However, in recent modifications of load theory, visual spatial working memory is argued to have similar effects as perceptual load [76–79]. Accordingly, load theory would seem to predict that the visual tracking task should decrease MMN rather than increase MMN, which is the opposite of the actual findings from the visual tracking task. Importantly, when we included the results of the visual tracking tasks [27, 28], there was evidence for heterogeneity among effect sizes (79% of total variance reflects variation in true effect size). However, this heterogeneity was mainly due to one study [28]. When this study was excluded, heterogeneity among effect sizes was small (<14%). These findings suggest two possibilities: First, visual tracking tasks may actually differ in their effects from that of other visual demands. Second, it is not visual tracking tasks per se but something specific in this study [28] that resulted in a different effect. Unfortunately, the (null) findings of the other study [27] are consistent with both possibilities. Similarly, another visual tracking study [70] did not find an effect of task load (i.e., tracking 1 of 2 compared to 3 of 6 circles) on the MMN. This study did not use the typical oddball task but used a regular pattern of tones as the standard and the violation of this pattern of tones as the deviant. Furthermore, close inspection of load theory [32, 33] suggests that theoretically, there is no clear reason why cognitive load in the present task design should increase attention to tones. According to load theory, attention is required to focus on the task-relevant stimuli in the context of task-irrelevant distracters. Cognitive load (in terms of working memory) reduces this attentional focus and thus, increases distraction [86]. This question has been most commonly tested with dual tasks in that a primary task that requires focused attention (e.g., Stroop) is combined with a secondary task that increases cognitive load (e.g., working memory) [87]. Because the task design in the visual tracking tasks does not fit this typical task design (e.g., [88]), it is unclear why visual tracking tasks should increase the MMN. Nonetheless, because the heterogeneity of effect sizes in the meta-analysis (which includes study [28]) provides some evidence that visual tracking tasks may increase the MMN, future studies are needed to determine whether or not the effects of visual tracking tasks on the MMN match those of other visual task demands.

The main limitation of the present study is that we conducted the meta-analysis only after conducting this study. Prior to designing this study, we approached the literature from a null hypothesis significance perspective, which suggested clear effects of visual task demands on the auditory MMN, as well as clear behavioral effects of perceptual load. We were therefore surprised when our results did not suggest any effects of perceptual load on the MMN, despite the fact that our sample size was at least twice as the average sample size in previous studies. However, in hindsight, our null findings are not entirely surprising if the results of our meta-analysis are used to estimate the power of our study. To estimate power, we assumed that the true effect size was captured in the meta-analysis of the five previous studies [22–26]. The mean difference was -0.50 μV (*k* = 5, *N* = 69). Because *t*(68) = -3.51, the corresponding effect size in terms of Cohen’s *d*_z_ was -0.42 (see equation 7 in [75]). With this effect size, a two-tailed alpha of .05, and a sample size of 28, power = .57 (estimated with G*Power, [89]). To obtain a power of .90, at least 62 subjects would have been required. Because of limited power, the present null findings do not provide convincing evidence for or against an effect of perceptual load on the MMN. From an estimation perspective [43], the limits of the 95% CI for the present results (between -0.91 and 0.85 μV) are quite wide and thus, provide only an imprecise estimate of the true effect size.

Critically, this apparent limitation in knowledge gain was apparent only after conducting the meta-analysis and was not obvious from the traditional narrative review. Because a narrative review has these limitations, a meta-analysis provides an excellent tool to integrate previous findings quantitatively [43]. As Gene V. Glass, a pioneer of meta-analysis in Psychology, wrote: “the proper integration of research requires the same statistical methods that are applied in primary data analysis” (p. 6, [90]). Indeed, it is surprising that researchers (like us) are extremely diligent to use statistical methods when analyzing their own data (i.e., primary data analysis) but use a narrative review when summarizing the previous literature. Because of the advantages of meta-analytic tools, several journals (e.g., Lancet) now require that researchers put their study in context by combining previous studies meta-analytically and providing a pooled estimate of the effect size [91, 92]. Although this places substantial demands on researchers, it provides a context in which to quantitatively evaluate the knowledge gained from the new study.

The results of the present meta-analysis are highly informative for future research in this area. If the true effect size is assumed to be captured by the present results together with previous studies that showed homogenous effect sizes [22–27], the mean difference was -0.43 μV (*k* = 7, *N* = 106). Because *t*(105) = -3.46, the corresponding effect size in terms of Cohen’s *d*_z_ was -0.34 (see equation 7 in [75]). With this effect size, a two-tailed alpha of .05, and a desired power of .90 [89], at least 93 subjects would be required.

Whereas power is used in significance testing, *precision* is used in estimation [42, 43]. Precision may be more informative than power because it can be used to estimate the expected size of the confidence intervals in meaningful units. Given *t*(105) = -3.46 and a mean difference of -0.43 μV, the *SD* of the difference scores was 1.28 (p. 200 in [93]). Using the *Precision one* page in ESCI [43], we determined that if 1.28 is assumed as the population *SD* and *n* = 93, then the expected average size of the margin of error would be 0.31 μV with 99% assurance. This means that in 99% of all replications, the 95% CI would extend no more than 0.31 μV above and below the observed mean effect.

The meta-analysis suggests that to obtain sufficient power, any future study probably needs to include at least 93 subjects. This study would need to run as many subjects as were run in all seven studies that were included in the meta-analysis (*k* = 7, *n* = 106) [22–27]. Unfortunately, there are three reasons to suggest an even larger sample size than *n* = 100. One reason is that the above precision analysis suggests that with 99% assurance (i.e., in 99% of replications), a reasonable estimate for the margin of error will be +/- 0.31 μV. Notably, the present meta-analysis (*k* = 7, *n* = 106) obtained a margin of error of 0.24 μV. Thus, the 95% CI is consistent with a true effect size of -0.18 μV (i.e., -0.43 +0.24). If this is the true effect size, then the 95% CI in a future study with *n* = 100 might well cross zero (i.e., -0.18 + 0.31 = +0.13). Therefore, a precision of 0.10 μV seems more desirable, but improving precision to 0.10 μV with 99% assurance would require more than 700 subjects. Such a task seems too demanding for an individual research lab, but may be tackled in a collaborative project [58].

Another reason to recommend a very large sample size is that in general, a meta-analysis of many small, published studies tends to overestimate the true effect size (for review, see [83]). In previous studies, sample sizes varied between 9 and 28 [22–27]. If the results of the meta-analysis (*k* = 7, *N* = 106) with Cohen’s *d*_z_ = -0.34 are assumed as the true effect size, then power for these studies ranged from .15 to .41. As reviewed in several recent papers [81–83], low power has several undesirable consequences. One of these consequences is that if significance is obtained in a low-powered study, then the true effect size is overestimated. This phenomenon is called the *winner’s curse* (for review, see [83]). The reason is that if a study has low power, then an observed effect needs to be much larger than the true effect to yield significance. Thus, in a meta-analysis of only small published studies, the individual studies may have inflated effect sizes, and this will inflate the estimated effect when these studies are combined in the meta-analysis. In contrast, large studies should be less biased when it comes to overestimating the true effect size [82, 83]. Simulations suggest that at a power of .30, effect size inflation may be 20% of the actual effect size (for review, see [83]). To adjust for any bias from inflated effect sizes, we conducted a power analysis in which we simply reduced the observed effect size (*d*_z_) by 20% from -0.34 to -0.27. For this effect size, 150 subjects would be required to obtain a power of .90 at two-tailed α = .05. In terms of precision, the estimated precision (at 99% assurance) would be 0.23 μV, similar to the observed precision in the present meta-analysis.

A third reason to recommend a large sample size is that small changes in data analytic strategies affect results more strongly in small than large studies. This is referred to as *vibration of effects* [55]. If researchers tend to select the most favorable results in their analyses, then the combined results from many small studies will suggest a stronger effect than the result from a single large study. Such vibration of effects is a potential problem in EEG research because electrodes and intervals are often not selected a priori but only after looking at the data. Because details of the data analyses are partly determined by the actual data, this circularity inflates effect sizes and has been referred to as double dipping [94], nonindependence error [95, 96], or baloney [97]. To eliminate this error, guidelines recommend selecting electrodes and intervals only on the basis of previous research [98]. In practice however, researchers often define electrodes and intervals from their own data as well as the previous literature. One reasonable explanation may be that because the physical parameters differ slightly between studies, the effects on the EEG might also differ slightly between studies. Nonetheless, this procedure is circular. Therefore, future studies would provide strong evidence if data processing and selection of electrodes and intervals are defined a priori and preregistered [99].

Taken together, the present meta-analyses suggest that future studies need to include a large sample size to improve knowledge gain. One approach would be to conduct this research collaboratively [58]. If this is not feasible, all individual studies should be published and made available for meta-analysis. However, as discussed recently [83], some areas in neuroscience may have conducted many small studies in the same field that, when taken together, used more subjects than would have seemed necessary to address the research question. This raises ethical considerations as to efficient use of resources.

The present research has at least five other limitations. First, although the meta-analysis supports the conclusion that demanding visual tasks decrease the MMN, the mechanism for this decrease is unresolved. The MMN has been proposed to reflect either sensory memory or neural adaptation (for review, see [3]). Because the traditional oddball task (as used in all studies in the meta-analysis) potentially confounds both processes, a *many standards* task (or more formally called *equiprobable sequence*) reduces effects of neural adaptation on the MMN [3, 100, 101]. The many standards task is run separately from an oddball task. As in the oddball task with a single deviant and single standard, the *control* stimulus is the same stimulus as the deviant and is presented with the same probability as in the oddball task. To illustrate with an example [102], in the oddball task, 500 Hz was the deviant (10%) and 550 Hz was the standard; and in the many standards task, 500 Hz was the control (10%). Thus, the deviant in the oddball task and the control in the many standards task were presented with equal probability. Different from the oddball task, however, the standard is replaced by several other stimuli so that across all stimuli (including the control), the probability for each stimulus is the same. Because all stimuli in the many standards task are shown with the same probability, there should be no sensory memory effect (in contrast to the oddball task). In the example study, the control (500 Hz) was presented together with nine other stimuli (between 550 and 1179 Hz) so that each stimulus had a probability of 10%. Furthermore, on average, these stimuli should physically differ more strongly from the control (in the many standards task) than the standard differs from the deviant (in the oddball task). In the example, the other nine stimuli (between 550 and 1179 Hz) differed more (on average) from the control than the standard (550 Hz). The reason is that if neural adaptation to these stimuli also leads to neural adaptation to the control (because the stimuli somewhat resemble the control), then this neural adaptation effect should be smaller in the many standards task than in the oddball task, as the physical difference between control and the other stimuli is larger than that between deviant and standard. Therefore, neural adaptation cannot account for the finding that the response is larger to the deviant than to the control. So, by computing the response to the deviant in the oddball task minus the physically identical control in the many-standards task, the resulting difference ERP should be mainly affected by sensory memory rather than neural adaption [101]. Future research may want to include this many-standards task, although confounding effects from neural adaptation and lateral inhibition may not be eliminated completely [3, 6]. Further, even if the many-standards task may be successful in isolating sensory memory effects on the MMN, it would be unresolved if these changes are caused specifically by changes in standard formation or deviance detection [3, 5, 29]. Last, although the mechanism for effects of attention on the MMN apparently involve corticofugal pathways, the exact neural mechanism has yet to be determined [29].

Second, our meta-analysis was highly selective in that we focused on studies that resembled the current study design as much as possible. That is, we included studies only if they were published, used EEG, the visual task demands were continuous and varied between high and low load within the same task, and the task-irrelevant tones were presented in a typical oddball task simultaneously with the visual stimuli. Because we included only published studies, our meta-analysis is mainly an attempt to quantitatively integrate previous research rather than to rely solely on a narrative review with a focus on significance. Thus, meta-analytic tools with estimation permit more accurate representations of the combined evidence in a field than a narrative review [43]. This is supported by successful meta-analyses of MMN-relevant studies [103–107], although this task may be impossible if methods vary too much [108]. Further, the focus on studies that resembled the current study design as much as possible has the advantage that our meta-analysis addresses a specific question. However, our selection criteria may not be shared by other researchers. For example, it may well be that effects of studies that manipulated visual demands with different tasks (rather than within levels of the same task) or that presented patterned auditory sequences (rather than a typical oddball task) do not differ from those in the present meta-analysis. These questions are beyond the scope of the present study, because a much more comprehensive meta-analysis would be needed to address these questions. Because the MMN has been studied extensively, there is an abundance of studies that could be used to evaluate effects of different paradigms and the role of potential moderators of the MMN. Such a future meta-analysis should also code for many study features (e.g., study quality, choice of deviance, task performance, EEG interval and electrodes) to test for moderating effects of these variables. Because the present meta-analysis was limited to a few studies, the analysis of moderators was not feasible.

Third, although the present study found strong behavioral effects of perceptual load, it could be argued that with stronger manipulations of perceptual load, even an effect on the MMN would be apparent. However, unless there is a criterion for a high level of perceptual load that is independent from its effects on the MMN, this reasoning is circular (as addressed above). However, a recent study [51] used the same visual stimuli in a visual search task and in a flanker task (i.e., perceptual load) and examined the correspondence of the behavioral effects between visual search task and flanker task. Results showed that inefficient visual search (as indicated by large search slopes) corresponded with high perceptual load. Because these results suggest that perceptual load may be defined independently (with reference to visual search), future studies may employ a similar set up to strengthen the claim for high perceptual load.

Fourth, the present results do not address gender differences. When we designed the present study, we reviewed previous studies and found no evidence that gender differences were a matter of concern [22–28]. For example, two studies used either only men [23] or mostly men (9 out of 11)[28]. Because at our university, both genders are not represented equally, we tried to run as many subjects as possible in the available time rather than to try to counterbalance gender. However, because gender differences on the MMN have been found in related areas [109–111], the present effects may be moderated by gender. At face value, however, there is no apparent pattern because the two studies with mostly men reported effects in opposite directions [23, 28]. Because large sample sizes are recommended for future studies, gender differences may need to be addressed in a meta-analysis. To facilitate such a meta-analysis, the raw data should be made available (provided for our study in the S1 File).

Fifth, the present study as well as the meta-analysis focused on a single electrode (Fz) and thus, did not consider laterality effects. Again, previous studies did not suggest laterality effects [22–28]. However, studies in other areas reported laterality effects for the MMN [2, 53, 112–114]. Thus, future studies will need to address potential laterality effects.

## Conclusions

We studied the effects of visual perceptual load on the MMN to simultaneous, auditory distracters in a typical oddball task. The results of the empirical study did not provide evidence for an effect of perceptual load on the MMN. A subsequent meta-analysis, however, suggested that the present (null) findings did not systematically differ from previous, similar studies that varied visual task demands. When the available evidence was combined, the meta-analysis confirmed that demanding visual tasks reduce the MMN to auditory distracters. However, because the meta-analysis was based on small studies and because of potential publication biases, future studies should have large samples (*n* > 150) to provide confirmatory evidence for the results of the present meta-analysis. Future studies should also use control conditions that reduce confounding effects of neural adaptation, use load manipulations that are defined independently and also address gender differences and laterality effects. Last, because the scope of the present meta-analysis was narrow, a more comprehensive meta-analysis is needed to determine whether or not the present results can be generalized to different task configurations (e.g., frequency vs. duration deviants, choice of electrodes, working memory tasks, intra- vs. intermodal). Because the MMN has been studied extensively, there is an abundance of studies that may be used to evaluate effects of different paradigms and the role of potential moderators of the MMN.

## Supporting Information

S1 File Data file in tab-delimited format. (TXT)

## References

[1] Roye A, Jacobsen T, Schröger E. Personal significance is encoded automatically by the human brain: an event-related potential study with ringtones. Eur J Neurosci. 2007;26(3):784–90. 10.1111/j.1460-9568.2007.05685.x 17634070

[2] Näätänen R, Paavilainen P, Rinne T, Alho K. The mismatch negativity (MMN) in basic research of central auditory processing: A review. Clin Neurophysiol. 2007;118(12):2544–90. 10.1016/j.clinph.2007.04.026 17931964

[3] Fishman YI. The Mechanisms and Meaning of the Mismatch Negativity. Brain Topogr. 2014;27(4):500–26. 10.1007/s10548-013-0337-3 24276221

[4] Duncan CC, Barry RJ, Connolly JF, Fischer C, Michie PT, Näätänen R, et al Event-related potentials in clinical research: Guidelines for eliciting, recording, and quantifying mismatch negativity, P300, and N400. Clin Neurophysiol. 2009;120(11):1883–908. 10.1016/j.clinph.2009.07.045 19796989

[5] Sussman ES, Chen S, Sussman-Fort J, Dinces E. The Five Myths of MMN: Redefining How to Use MMN in Basic and Clinical Research. Brain Topogr. 2014;27(4):553–64. 10.1007/s10548-013-0326-6 24158725 PMC4000291

[6] May PJC, Tiitinen H. Mismatch negativity (MMN), the deviance-elicited auditory deflection, explained. Psychophysiology. 2010;47(1):66–122. 10.1111/j.1469-8986.2009.00856.x 19686538

[7] Woldorff MG, Hillyard SA. Modulation of early auditory processing during selective listening to rapidly presented tones. Electroencephalogr Clin Neurophysiol. 1991;79(3):170–91. 10.1016/0013-4694(91)90136-r 1714809

[8] Näätänen R. The role of attention in auditory information-processing as revealed by event-related potentials and other brain measures of cognitive function. Behav Brain Sci. 1990;13(2):201–32.

[9] Alho K, Woods DL, Algazi A, Näätänen R. Intermodal selective attention. II. Effects of attentional load on processing of auditory and visual stimuli in central space. Electroencephalogr Clin Neurophysiol. 1992;82:356–68. 1374704 10.1016/0013-4694(92)90005-3

[10] Dyson BJ, Alain C, He Y. Effects of visual attentional load on low-level auditory scene analysis. Cogn Affect Behav Neurosci. 2005;5(3):319–38. 10.3758/cabn.5.3.319 16396093

[11] Alho K, Woods DL, Algazi A. Processing of auditory stimuli during auditory and visual attention as revealed by event-related potentials. Psychophysiology. 1994;31:469–79. 7972601 10.1111/j.1469-8986.1994.tb01050.x

[12] Dittmann-Balcar A, Thienel R, Schall U. Attention-dependent allocation of auditory processing resources as measured by mismatch negativity. Neuroreport. 1999;10(18):3749–53. 10.1097/00001756-199912160-00005 10716203

[13] Harmony T, Bernal J, Fernandez T, Silva-Pereyra J, Fernandez-Bouzas A, Marosi E, et al Primary task demands modulate P3a amplitude. Brain Res Cogn Brain Res. 2000;9(1):53–60. 10.1016/s0926-6410(99)00044-0 10666557

[14] Kathmann N, Frodl-Bauch T, Hegerl U. Stability of the mismatch negativity under different stimulus and attention conditions. Clin Neurophysiol. 1999;110:317–23. 10210621 10.1016/s1388-2457(98)00011-x

[15] Otten LJ, Alain C, Picton TW. Effects of visual attentional load on auditory processing. Neuroreport. 2000;11(4):875–80. 10.1097/00001756-200003200-00043 10757537

[16] Müller BW, Achenbach C, Oades RD, Bender S, Schail U. Modulation of mismatch negativity by stimulus deviance and modality of attention. Neuroreport. 2002;13:1317–20. 12151795 10.1097/00001756-200207190-00021

[17] Sussman ES, Bregman AS, Wang WJ, Khan FJ. Attentional modulation of electrophysiological activity in auditory cortex for unattended sounds within multistream auditory environments. Cogn Affect Behav Neurosci. 2005;5(1):93–110. 15913011 10.3758/cabn.5.1.93

[18] Takegata R, Brattico E, Tervaniemi M, Varyagina O, Näätänen R, Winkler I. Preattentive representation of feature conjunctions for concurrent spatially distributed auditory objects. Brain Res Cogn Brain Res. 2005;25(1):169–79. 10.1016/j.cogbrainres.2005.05.006 15953710

[19] Wei JH, T.C. C, Luo YJ. A modified oddball paradigm "cross-modal delayed response" and the research on mismatch negativity. Brain Res Bull. 2002;57(2):221–30. 11849829 10.1016/s0361-9230(01)00742-0

[20] Muller-Gass A, Stelmack RM, Campbell KB. "…and were instructed to read a self-selected book while ignoring the auditory stiimuli": The effects of task demands on the mismatch negativity. Clin Neurophysiol. 2005;116(9):2142–52. 10.1016/j.clinph.2005.05.012 16029961

[21] Muller-Gass A, Stelmack RM, Campbell KB. The effect of visual task difficulty and attentional direction on the detection of acoustic change as indexed by the Mismatch Negativity. Brain Res. 2006;1078(1):112–30. 10.1016/j.brainres.2005.12.125 16497283

[22] Haroush K, Hochstein S, Deouell LY. Momentary fluctuations in allocation of attention: Cross-modal effects of visual task load on auditory discrimination. J Cogn Neurosci. 2010;22(7):1440–51. 10.1162/jocn.2009.21284 19580389

[23] Kramer AF, Trejo LJ, Humphrey D. Assessment of mental workload with task-irrelevant auditory probes. Biol Psychol. 1995;40(1–2):83–100. 10.1016/0301-0511(95)05108-2 7647188

[24] Singhal A, Doerfling P, Fowler B. Effects of a dual task on the N100-P200 complex and the early and late Nd attention waveforms. Psychophysiology. 2002;39(2):236–45. 10.1017/s0048577201392156 12212674

[25] Yucel G, Petty C, McCarthy G, Belger A. Graded visual attention modulates brain responses evoked by task-irrelevant auditory pitch changes. J Cogn Neurosci. 2005;17(12):1819–28. 10.1162/089892905775008698 16356321

[26] Yucel G, Petty C, McCarthy G, Belger A. Visual task complexity modulates the brain's response to unattended auditory novelty. Neuroreport. 2005;16(10):1031–6. 10.1097/00001756-200507130-00001 15973143

[27] Muller-Gass A, Macdonald M, Schröger E, Sculthorpe L, Campbell K. Evidence for the auditory P3a reflecting an automatic process: Elicitation during highly-focused continuous visual attention. Brain Res. 2007;1170:71–8. 10.1016/j.brainres.2007.07.023 17692834

[28] Zhang P, Chen XC, Yuan P, Zhang DR, He S. The effect of visuospatial attentional load on the processing of irrelevant acoustic distractors. Neuroimage. 2006;33(2):715–24. 10.1016/j.neuroimage.2006.07.015 16956775

[29] Campbell TA. A theory of attentional modulations of the supratemporal generation of the auditory mismatch negativity (MMN). Front Hum Neurosci. 2015;8 10.3389/fnhum.2014.01065 PMC4310267 25688198

[30] Pannese A, Herrmann CS, Sussman E. Analyzing the Auditory Scene: Neurophysiologic Evidence of a Dissociation Between Detection of Regularity and Detection of Change. Brain Topogr. 2015;28(3):411–22. 10.1007/s10548-014-0368-4 24771006 PMC4210364

[31] Lavie N. Attention, distraction, and cognitive control under load. Curr Dir Psychol. 2010;19(3):143–8. 10.1177/0963721410370295

[32] Lavie N, Tsal Y. Perceptual load as a major determinant of the locus of selection in visual-attention. Percept Psychophys. 1994;56(2):183–97. 10.3758/bf03213897 7971119

[33] Lavie N, Hirst A, de Fockert JW, Viding E. Load theory of selective attention and cognitive control. J Exp Psychol-Gen. 2004;133(3):339–54. 10.1037/0096-3445.133.3.339 15355143

[34] Raveh D, Lavie N. Load-induced inattentional deafness. Atten Percept Psychophys. 2014 10.3758/s13414-014-0776-2 PMC4677383 25287617

[35] Oostenveld R, Fries P, Maris E, Schoffelen JM. FieldTrip: Open source software for advanced analysis of MEG, EEG, and invasive electrophysiological data. Comput Intell Neurosci. 2011;2011:156869 10.1155/2011/156869 21253357 PMC3021840

[36] Wiens S, Syrjänen E. Directed attention reduces processing of emotional distracters irrespective of valence and arousal level. Biol Psychol. 2013;94(1):44–54. 10.1016/j.biopsycho.2013.05.001 23669534

[37] Luck SJ. An introduction to the event-related potential technique Cambridge, MA: MIT Press; 2005.

[38] Fischer C, Morlet D, Bouchet P, Luaute J, Jourdan C, Salord F. Mismatch negativity and late auditory evoked potentials in comatose patients. Clin Neurophysiol. 1999;110(9):1601–10. 10.1016/s1388-2457(99)00131-5 10479027

[39] Fischer C, Luaute J, Morlet D. Event-related potentials (MMN and novelty P3) in permanent vegetative or minimally conscious states. Clin Neurophysiol. 2010;121(7):1032–42. 10.1016/j.clinph.2010.02.005 20202899

[40] Macmillan NA, Creelman CD. Detection theory: A user's guide 2nd ed. Mahway, New Jersey: Laurence Erlbaum Associates 2005.

[41] Snodgrass JG, Corwin J. Pragmatics of measuring recognition memory: Applications to dementia and amnesia. J Exp Psychol Gen. 1988;117(1):34–50. 2966230 10.1037//0096-3445.117.1.34

[42] Cumming G. The New Statistics: Why and how. Psychol Sci. 2014;25(1):7–29. 10.1177/0956797613504966 24220629

[43] Cumming G. Understanding the new statistics: Effect sizes, confidence intervals, and meta-analysis New York: Routledge; 2012.

[44] Hoekstra R, Morey RD, Rouder JN, Wagenmakers E-J. Robust misinterpretation of confidence intervals. Psychon Bull Rev. 2014:1–8. 24420726 10.3758/s13423-013-0572-3

[45] Morey R, Hoekstra R, Rouder J, Lee M, Wagenmakers E-J. The fallacy of placing confidence in confidence intervals. Psychon Bull Rev. 2015:1–21. 26450628 10.3758/s13423-015-0947-8 PMC4742505

[46] Dienes Z. Understanding psychology as a science: An introduction to scientific and statistical inference New York: Palgrave Macmillan; 2008.

[47] Pfister R, Janczyk M. Confidence intervals for two sample means: Calculation, interpretation, and a few simple rules. Adv Cogn Psychol. 2013;9(2):74–80. Epub 2013/07/05. 10.2478/v10053-008-0133-x 23826038 PMC3699740

[48] Rosnow RL, Rosenthal R. Some things you learn aren't so—Cohens paradox, Aschs paradigm, and the interpretation of interaction. Psychol Sci. 1995;6(1):3–9. 10.1111/j.1467-9280.1995.tb00297.x

[49] Näätänen R, Kreegipuu K. The Mismatch Negativity (MMN) In: Kappenman ES, Luck SJ, editors. Oxford Handbook of Event-Related Potential Components. Oxford: Oxford University Press; 2011.

[50] Näätänen R, Gaillard AWK. 5 The Orienting Reflex and the N2 Deflection of the Event-Related Potential (ERP) In: Anthony WKG, Walter R, editors. Advances in Psychology. Volume 10: North-Holland; 1983 p. 119–41.

[51] Roper ZJJ, Cosman JD, Vecera SP. Perceptual load corresponds with factors known to influence visual search. J Exp Psychol Hum Percept Perform. 2013;39(5):1340–51. 10.1037/a0031616 23398258 PMC3928141

[52] Sand A, Wiens S. Processing of unattended, simple negative pictures resists perceptual load. Neuroreport. 2011;22(7):348–52. 10.1097/WNR.0b013e3283463cb1 21464776

[53] Deouell LY. The frontal generator of the mismatch negativity revisited. J Psychophysiol. 2007;21(3–4):188–203. 10.1027/0269-8803.21.34.188

[54] Bakker M, van Dijk A, Wicherts JM. The rules of the game called Psychological Science. Perspect Psychol Sci. 2012;7(6):543–54. 10.1177/1745691612459060 26168111

[55] Ioannidis JPA. Why most published research findings are false. PLoS Med. 2005;2(8):696–701. 10.1371/journal.pmed.0020124 PMC1182327 16060722

[56] Ioannidis JPA. Why Science Is Not Necessarily Self-Correcting. Perspect Psychol Sci. 2012;7(6):645–54. 10.1177/1745691612464056 26168125

[57] Calin-Jageman RJ, Caldwell TL. Replication of the superstition and performance study by Damisch, Stoberock, and Mussweiler (2010). Soc Psychol. 2014;45(3):239–45. 10.1027/1864-9335/a000190

[58] Klein RA, Ratliff KA, Vianello M, Adams RB, Bahnik S, Bernstein MJ, et al Investigating Variation in Replicability A "Many Labs'' Replication Project. Soc Psychol. 2014;45(3):142–52. 10.1027/1864-9335/a000178

[59] Parks NA, Hilimire MR, Corballis PM. Steady-state Signatures of Visual Perceptual Load, Multimodal Distractor Filtering, and Neural Competition. J Cogn Neurosci. 2011;23(5):1113–24. 10.1162/jocn.2010.21460 20146614

[60] Chait M, Ruff CC, Griffiths TD, McAlpine D. Cortical responses to changes in acoustic regularity are differentially modulated by attentional load. Neuroimage. 2012;59(2):1932–41. 10.1016/j.neuroimage.2011.09.006 21945789 PMC3271381

[61] Klemen J, Büchel C, Rose M. Perceptual load interacts with stimulus processing across sensory modalities. Eur J Neurosci. 2009;29(12):2426–34. 10.1111/j.1460-9568.2009.06774.x 19490081

[62] Rinne T. Activations of human auditory cortex during visual and auditory selective attention tasks with varying difficulty. Open Neuroimag J. 2010;4:187–93. 10.2174/1874440001004010187 21760872 PMC3134945

[63] Sussman ES, Winkler I, Schröger E. Top-down control over involuntary attention switching in the auditory modality. Psychon Bull Rev. 2003;10(3):630–7. 10.3758/bf03196525 14620357

[64] Regenbogen C, De Vos M, Debener S, Turetsky BI, Mossnang C, Finkelmeyer A, et al Auditory processing under cross-modal visual load investigated with simultaneous EEG-fMRI. PLoS One. 2012;7(12):e52267 10.1371/journal.pone.0052267 23251704 PMC3522643

[65] Berti S, Schröger E. Working memory controls involuntary attention switching: evidence from an auditory distraction paradigm. Eur J Neurosci. 2003;17(5):1119–22. 10.1046/j.1460-9568.2003.02527.x 12653989

[66] SanMiguel I, Corral MJ, Escera C. When loading working memory reduces distraction: Behavioral and electrophysiological evidence from an auditory-visual distraction paradigm. J Cogn Neurosci. 2008;20(7):1131–45. 10.1162/jocn.2008.20078 18284343

[67] Restuccia D, Della Marca G, Marra C, Rubino M, Valeriani M. Attentional load of the primary task influences the frontal but not the temporal generators of mismatch negativity. Brain Res Cogn Brain Res. 2005;25(3):891–9. 10.1016/j.cogbrainres.2005.09.023 16289727

[68] Erlbeck H, Kubler A, Kotchoubey B, Veser S. Task instructions modulate the attentional mode affecting the auditory MMN and the semantic N400. Front Hum Neurosci. 2014;8:654 10.3389/fnhum.2014.00654 25221494 PMC4145469

[69] Rissling AJ, Park SH, Young JW, Rissling MB, Sugar CA, Sprock J, et al Demand and modality of directed attention modulate "pre-attentive" sensory processes in schizophrenia patients and nonpsychiatric controls. Schizophr Res. 2013;146(1–3):326–35. 10.1016/j.schres.2013.01.035 23490760 PMC3622836

[70] Sculthorpe LD, Collin CA, Campbell KB. The influence of strongly focused visual attention on the detection of change in an auditory pattern. Brain Res. 2008;1234:78–86. 10.1016/j.brainres.2008.07.031 18674520

[71] Murphy JR, Rawdon C, Kelleher I, Twomey D, Markey PS, Cannon M, et al Reduced duration mismatch negativity in adolescents with psychotic symptoms: further evidence for mismatch negativity as a possible biomarker for vulnerability to psychosis. BMC Psychiatry. 2013;13(45):1–7. 10.11186/1471-244X-13-45 23375130 PMC3598448

[72] Sculthorpe LD, Ouellet DR, Campbell KB. MMN elicitation during natural sleep to violations of an auditory pattern. Brain Res. 2009;1290:52–62. 10.1016/j.brainres.2009.06.013 19527697

[73] Morlet D, Fischer C. MMN and Novelty P3 in Coma and Other Altered States of Consciousness: A Review. Brain Topogr. 2014;27(4):467–79. 10.1007/s10548-013-0335-5 24281786 PMC5034015

[74] Cumming G, Finch S. Inference by eye: Confidence intervals and how to read pictures of data. Am Psychol. 2005;60(2):170–80. 10.1037/0003-066x.60.2.170 15740449

[75] Lakens D. Calculating and reporting effect sizes to facilitate cumulative science: a practical primer for t-tests and ANOVAs. Front Psychol. 2013;4:863 10.3389/fpsyg.2013.00863 24324449 PMC3840331

[76] Konstantinou N, Bahrami B, Rees G, Lavie N. Visual short-term memory load reduces retinotopic cortex response to contrast. J Cogn Neurosci. 2012;24(11):2199–210. 10.1162/jocn_a_00279 22905823

[77] Konstantinou N, Beal E, King JR, Lavie N. Working memory load and distraction: dissociable effects of visual maintenance and cognitive control. Atten Percept Psychophys. 2014 10.3758/s13414-014-0742-z PMC4212201 25085738

[78] Konstantinou N, Lavie N. Dissociable roles of different types of working memory load in visual detection. J Exp Psychol Hum Percept Perform. 2013;39(4):919–24. 10.1037/a0033037 23713796 PMC3725889

[79] Roper ZJJ, Vecera SP. Visual short-term memory load strengthens selective attention. Psychon Bull Rev. 2014;21(2):549–56. 10.3758/s13423-013-0503-3 24002967

[80] Sussman ES. A new view on the MMN and attention debate: the role of context in processing auditory events. J Psychophysiol. 2007;21(3). 10.1027/0269-8803.21.3.xxx

[81] Yarkoni T. Big correlations in little studies: Inflated fMRI correlations reflect low statistical power-Commentary on Vul et al. (2009). Perspect Psychol Sci. 2009;4(3):294–8. 10.1111/j.1745-6924.2009.01127.x 26158966

[82] Ingre M. Why small low-powered studies are worse than large high-powered studies and how to protect against "trivial" findings in research: Comment on Friston (2012). Neuroimage. 2013;81:496–8. 10.1016/j.neuroimage.2013.03.030 23583358

[83] Button KS, Ioannidis JPA, Mokrysz C, Nosek BA, Flint J, Robinson ESJ, et al Power failure: why small sample size undermines the reliability of neuroscience. Nat Rev Neurosci. 2013;14(5):365–76. 10.1038/nrn3475 23571845

[84] Sterne JAC, Sutton AJ, Ioannidis JPA, Terrin N, Jones DR, Lau J, et al Recommendations for examining and interpreting funnel plot asymmetry in meta-analyses of randomised controlled trials. Br Med J. 2011;343 10.1136/bmj.d4002 21784880

[85] Borenstein M, Hedges LV, Higgins JPT, Rothstein HR. Introduction to Meta-Analysis: John Wiley & Sons, Ltd; 2009.

[86] Lavie N. Distracted and confused?: selective attention under load. Trends Cogn Sci. 2005;9(2):75–82. 10.1016/j.tics.2004.12.004 15668100

[87] de Fockert JW, Rees G, Frith CD, Lavie N. The role of working memory in visual selective attention. Science. 2001;291(5509):1803–6. 11230699 10.1126/science.1056496

[88] Rose M, Schmid C, Winzen A, Sommer T, Büchel C. The functional and temporal characteristics of top-down modulation in visual selection. Cereb Cortex. 2005;15(9):1290–8. 15616129 10.1093/cercor/bhi012

[89] Faul F, Erdfelder E, Lang AG, Buchner A. G*Power 3: A flexible statistical power analysis program for the social, behavioral, and biomedical sciences. Behav Res Methods. 2007;39(2):175–91. 10.3758/bf03193146 17695343

[90] Glass GV. Primary, Secondary, and Meta-Analysis of Research. Educ Res. 1976;5(10):3–8. 10.3102/0013189x005010003

[91] Kleinert S, Benham L, Collingridge D, Summerskill W, Horton R. Further emphasis on research in context. Lancet. 2014;384(9961):2176–7. 10.1016/S0140-6736(14)62047-X 25625383

[92] Clarke M, Hopewell S, Chalmers I. Clinical trials should begin and end with systematic reviews of relevant evidence: 12 years and waiting. Lancet. 2010;376(9734):20–1. 10.1016/s0140-6736(10)61045-8 20609983

[93] Howell DC. Statistical methods for psychology 8 ed. U.S.A.: Wadsworth, Cengage Learning; 2013.

[94] Kriegeskorte N, Simmons WK, Bellgowan PSF, Baker CI. Circular analysis in systems neuroscience: the dangers of double dipping. Nat Neurosci. 2009;12(5):535–40. 10.1038/nn.2303 19396166 PMC2841687

[95] Vul E, Harris C, Winkielman P, Pashler H. Puzzlingly High Correlations in fMRI Studies of Emotion, Personality, and Social Cognition. Perspect Psychol Sci. 2009;4(3):274–90. 10.1111/j.1745-6924.2009.01125.x 26158964

[96] Vul E, Harris C, Winkielman P, Pashler H. Reply to Comments on "Puzzlingly High Correlations in fMRI Studies of Emotion, Personality, and Social Cognition". Perspect Psychol Sci. 2009;4(3):319–24. 10.1111/j.1745-6924.2009.01132.x 26158970

[97] Cureton EE. Validity, reliability, and baloney. Educ Psychol Meas. 1950;10(1):94–6. 10.1177/001316445001000107

[98] Keil A, Debener S, Gratton G, Junghöfer M, Kappenman ES, Luck SJ, et al Committee report: Publication guidelines and recommendations for studies using electroencephalography and magnetoencephalography. Psychophysiology. 2014;51(1):1–21. 10.1111/psyp.12147 24147581

[99] Nosek BA, Lakens D. Registered Reports A Method to Increase the Credibility of Published Results. Soc Psychol. 2014;45(3):137–41. 10.1027/1864-9335/a000192

[100] Kimura M, Katayama J, Ohira H, Schröger E. Visual mismatch negativity: New evidence from the equiprobable paradigm. Psychophysiology. 2009;46(2):402–9. 10.1111/j.1469-8986.2008.00767.x 19207207

[101] Kujala T, Tervaniemi M, Schröger E. The mismatch negativity in cognitive and clinical neuroscience: Theoretical and methodological considerations. Biol Psychol. 2007;74(1):1–19. 10.1016/j.biopsycho.2006.06.001 16844278

[102] Jacobsen T, Schröger E. Is there pre-attentive memory-based comparison of pitch? Psychophysiology. 2001;38(4):723–7. 10.1017/s0048577201000993 11446587

[103] Umbricht D, Krljes S. Mismatch negativity in schizophrenia: a meta-analysis. Schizophr Res. 2005;76(1):1–23. 10.1016/j.schres.2004.12.002 15927795

[104] Alho K, Rinne T, Herron TJ, Woods DL. Stimulus-dependent activations and attention-related modulations in the auditory cortex: A meta-analysis of fMRI studies. Hear Res. 2014;307:29–41. 10.1016/j.heares.2013.08.001 23938208

[105] Bodatsch M, Brockhaus-Dumke A, Klosterkotter J, Ruhrmann S. Forecasting Psychosis by Event-Related Potentials-Systematic Review and Specific Meta-Analysis. Biol Psychiatry. 2015;77(11):951–8. 10.1016/j.biopsych.2014.09.025 25636178

[106] Cheng CH, Hsu WY, Lin YY. Effects of physiological aging on mismatch negativity: A meta-analysis. Int J Psychophysiol. 2013;90(2):165–71. 10.1016/j.ijpsycho.2013.06.026 23831479

[107] Daltrozzo J, Wioland N, Mutschler V, Kotchoubey B. Predicting coma and other low responsive patients outcome using event-related brain potentials: A meta-analysis. Clin Neurophysiol. 2007;118(3):606–14. 10.1016/j.clinph.2006.11.019 17208048

[108] Bartha-Doering L, Deuster D, Giordano V, Zehnhoff-Dinnesen AA, Dobel C. A systematic review of the mismatch negativity as an index for auditory sensory memory: From basic research to clinical and developmental perspectives. Psychophysiology. 2015;52(9):1115–30. 10.1111/psyp.12459 26096130

[109] Ikezawa S, Nakagome K, Mimura M, Shinoda J, Itoh K, Homma I, et al Gender differences in lateralization of mismatch negativity in dichotic listening tasks. Int J Psychophysiol. 2008;68(1):41–50. 10.1016/j.ijpsycho.2008.01.006 18295364

[110] Matsubayashi J, Kawakubo Y, Suga M, Takei Y, Kumano S, Fukuda M, et al The influence of gender and personality traits on individual difference in auditory mismatch: A magnetoencephalographic (MMNm) study. Brain Res. 2008;1236:159–65. 10.1016/j.brainres.2008.07.120 18725215

[111] Kasai K, Nakagome K, Iwanami A, Fukuda M, Itoh K, Koshida I, et al No effect of gender on tonal and phonetic mismatch negativity in normal adults assessed by a high-resolution EEG recording. Brain Res Cogn Brain Res. 2002;13(3):305–12. 10.1016/s0926-6410(01)00125-2 11918996

[112] Paavilainen P, Alho K, Reinikainen K, Sams M, Näätänen R. Right-hemisphere dominance of different mismatch negativities. Electroencephalogr Clin Neurophysiol. 1991;78(6):466–79. 10.1016/0013-4694(91)90064-b 1712282

[113] Rinne T, Degerman A, Alho K. Superior temporal and inferior frontal cortices are activated by infrequent sound duration decrements: An fMRI study. Neuroimage. 2005;26(1):66–72. 10.1016/j.neuroimage.2005.01.017 15862206

[114] Levänen S, Ahonen A, Hari R, McEvoy L, Sams M. Deviant auditory stimuli activate human left and right auditory cortex differently. Cereb Cortex. 1996;6(2):288–96. 10.1093/cercor/6.2.288 8670657

